# Assessment of Knowledge, Attitude, and Practice of Iranian Nurses towards Toothbrush Maintenance and Use

**DOI:** 10.1155/2021/3694141

**Published:** 2021-12-14

**Authors:** Mohammad Nazarianpirdosti, Maryam Janatolmakan, Bahare Andayeshgar, Alireza khatony

**Affiliations:** ^1^Student Research Committee, Kermanshah University of Medical Sciences, Kermanshah, Iran; ^2^Social Development and Health Promotion Research Centre, Health Institute, Kermanshah University of Medical Sciences, Kermanshah, Iran; ^3^School of Health, Kermanshah University of Medical Sciences, Kermanshah, Iran; ^4^Infectious Diseases Research Center, Kermanshah University of Medical Sciences, Kermanshah, Iran

## Abstract

**Background:**

Since nurses are considered a role model in society, they should have sufficient knowledge, attitude, and practice in the field of oral hygiene. This study was aimed to assess the nurses' knowledge, practice, and attitude towards toothbrush maintenance and use.

**Methods:**

In this cross-sectional study, 325 nurses working in hospitals affiliated to Kermanshah University of Medical Sciences were randomly recruited. Data collection tools included a demographic information form and a researcher-made questionnaire on knowledge, attitude, and practice regarding toothbrush maintenance and use. Data were analyzed by SPSS software using descriptive and inferential statistics (Mann–Whitney *U* and Kruskal–Wallis H).

**Results:**

The mean scores of nurses' knowledge, attitude, and practice were 59.2 ± 16.4, 64.2 ± 20.6, and 51.4 ± 17.0 out of 100, respectively. There was no statistically significant relation between nurses' knowledge, attitude, and practice and their gender, age, level of education, and work experience.

**Conclusions:**

Nurses had moderate knowledge, attitude, and practice regarding toothbrush maintenance and use, which is not very desirable given their role model. Therefore, training courses are recommended to be held to increase the nurses' knowledge, attitude, and practice regarding toothbrush maintenance and use.

## 1. Background

Brushing is one of the most important and effective self-care methods which prevents oral diseases [1–3]. In addition, brushing reduces dental plaque and thus prevents decay and related diseases [4–8]. Therefore, it is highly important for nurses to have adequate knowledge about the correct brushing method and also toothbrush maintenance, replacement, and cleaning [9–12]. If the toothbrush is not properly maintained and used, it can cause oral infections and diseases [9–15]. Changing the toothbrush every 2.5–6 months and brushing for two minutes or more twice a day are some correct methods of toothbrush use [13, 16–22].

Today, as the largest group in the health sector, nurses have an important role in promoting health policies in the field of oral health [23–27]. Therefore, adequate knowledge and practice and positive attitude regarding toothbrush maintenance and use is of special importance for nurses [28–32]. Surveys in Europe and the United States show that nurses consider oral health one of the most important nursing practices [1, 33]. A study in Malaysia showed that despite the limited knowledge of nurses about oral health, they had a good attitude to it [34]. The results of a study in India (2018) on oral health showed that 70% of nurses had poor knowledge, 83% had a positive attitude, and 69% had poor practice [30]. A study also showed that 82% of Iranian nurses had poor oral care practice [35]. The results of a study in Norway (2012) showed that 80% of nurses considered oral health an important issue, while 9.1% found patient oral care unpleasant [36]. In another study, the knowledge of Nigerian nurses and midwives regarding oral health was inadequate [37]. The results of a study on Australian nurses showed that 74.0% of them were aware of the important oral health practices [38].

Considering the educational role of nurses and the lack of information about the knowledge, attitude, and practice of the nurses of Kermanshah University of Medical Sciences regarding toothbrush maintenance and use, the current study was conducted to shed more light on this lacuna. This study sought to answer the following questions:

1. What is the nurses' level of knowledge about toothbrush maintenance and use?
2. What is the nurses' attitude and practice regarding toothbrush maintenance and use?
3. What is the relationship between nurses' knowledge, attitude, and practice in toothbrush maintenance and use and their demographic variables?

## 2. Materials and Methods

### 2.1. Study Design

The present descriptive-analytical cross-sectional study was conducted from March to May 2019. The study was performed based on STROBE reporting criteria.

### 2.2. Sample and Sampling Method

The study population (*n* = 2042) consisted of nurses working in hospitals affiliated to Kermanshah University of Medical Sciences (7 hospitals). The sample size was estimated to be 325 using Cochran's formula and the results of the study of Sharif et al. with 95% confidence and the first type error equal to 5% [34]. The inclusion criteria consisted of employment in the field of nursing and consent to participation in the study.

### 2.3. Instruments

The study tools included a personal information form and a questionnaire on nurses' knowledge, attitude, and practice in toothbrush maintenance and use. The personal information form included 5 questions on gender, age, level of education, marital status, and work experience.

A valid and reliable questionnaire was used to assess the nurses' knowledge, attitude, and practice in toothbrush maintenance and use. This questionnaire was developed and validated by Janatolmakan et al. and had good psychometric properties. They examined the validity of the questionnaire by the qualitative and quantitative content validity method. In the qualitative section, the judgment of experts has been used, and in the quantitative section, the content validity index has been calculated, which has been equal to 0.87, 0.89, and 0.88 for the sections of knowledge, attitude, and practice, respectively. The reliability of the instrument was also tested and confirmed by the test-retest method. The correlation coefficients for the scales of knowledge, attitude, and practice were 0.87, 0.88, and 0.86, respectively [39].

The first part of the questionnaire was allocated to the assessment of knowledge and consisted of 10 multiple choice questions. Some of the questions in this section were as follows: “What is the right water temperature for brushing?” “What is the best way to brush?” and “When should the toothbrush be washed?”

To score this section, the correct and incorrect answers were given a score of one and zero, respectively. The range of scores was between 0 and 10, which was calculated on the basis of 100 and was divided as poor (≤49), medium (50–74), and good (≥75) knowledge.

The second part, with 6 questions, was allocated to evaluate the nurses' attitudes toward toothbrush maintenance and use. The items in this section were of two-choice type, and the answers included “agree and disagree.” Two of the items in this section were “The harder the toothbrush material, the better its function” and “foreign toothbrushes are more durable.” To calculate the scores, the answers “I agree” and “I disagree” were given one and zero points, respectively. The range of scores was between 0 and 6, which was calculated on the basis of 100 and was divided as negative (≤49) and positive (≥50) attitude.

The third section consisted of 10 multiple-choice questions to evaluate the nurses' practice in toothbrush maintenance and use. Some of the questions in this section were “What type of toothbrush do you use?” “Where do you keep your toothbrush” and “When do you wash your toothbrush?” To calculate the score of this section, scores 1 and 0 were assigned to the correct and incorrect answers, respectively. The range of scores was between 0 and 10, which was calculated on the basis of 100 and expressed as poor (≤49), moderate (50–74), and good (≥75) practice.

### 2.4. Data Collection

After receiving the approval of the university ethics committee, the researcher attended the nurses' place of work according to the work schedule. First, the objectives of the study were explained to the nurses, and if they willing to participate in the study, the questionnaires were provided to them. To ensure the validity of the data, the participants were given enough time to complete the questionnaires.

### 2.5. Data Analysis

Data were analyzed by SPSS-16 software using descriptive and inferential statistics. Mean, standard deviation, median, and simple and relative frequency distributions were used for the descriptive statistics section. In the inferential statistics section, Mann–Whitney *U*, Kolmogorov–Smirnov, and Kruskal–Wallis tests were used. The Kolmogorov–Smirnov test was used to evaluate the normality of the distribution of knowledge, attitude, and practice variables. The results showed that these variables had an abnormal distribution. The Mann–Whitney *U* test was used to examine the relationship between knowledge, attitude, and practice variables and gender and education level variables. The Kruskal–Wallis test was also used to examine the relationship between knowledge, attitude, and practice variables and age and work experience variables. The level of significance was set at <0.05.

### 2.6. Ethical Considerations

The Ethics Committee of Kermanshah University of Medical Sciences approved the study with the code IR.KUMS.REC.1397.874. Written informed consent was obtained from all participants. All participants were assured that their information and responses would be kept confidential. The protocol of the experiment was entirely in accordance to guidelines of national/international/institutional or Declaration of Helsinki.

## 3. Results

The mean age and work experience of the participants were 31.6 ± 5.6 and 5.3. ±2.7 years, respectively. Most of the participants were female (*n* = 197, 60.4%), single (*n* = 179, 55.1%), in the age range of 32–22 (*n* = 215, 66.2%) and had a bachelor's degree (*n* = 299, 92%) (Table 1).

**Figure 1:**
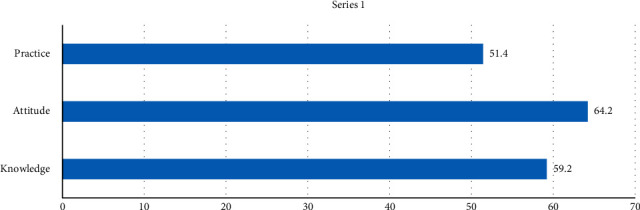
Comparison of nurses' knowledge, attitude and practice in toothbrush maintenance and use.

The mean score of nurses' knowledge about toothbrush maintenance and use was 59.2 ± 16.4 out of 100. The mean scores of nurses' attitude and practice were 64.2 ± 20.6 and 51.4 ± 17.0 out of 100, respectively (Figure 1 and Table 2). There was no statistically significant relationship between the mean scores of nurses' knowledge, attitude, and practice variables and gender, education, age, and work experience variables (Tables 3–5).

## 4. Discussion

This study aimed to investigate the Iranian nurses' knowledge, attitude, and practice regarding toothbrush maintenance and use. In the present study, most of the participants had a moderate level of knowledge and practice and an unfavorable attitude about the maintenance and use of toothbrushes. Proper maintenance and use of toothbrushes is an important part of oral hygiene [39]. Studies have reported that nurses have different levels of knowledge and practice about oral health. In this regard, Ibrahim et al. indicated that more than 90% of Sudanese nurses had good knowledge regarding oral health [2]. Furthermore, Sreenivasan et al. reported more than 80% of Indian nurses had good knowledge in this regard [33]. However, Ahmed et al. found 70% of Indian nurses had poor knowledge about oral health [35]. In two studies conducted on the Indian and Iranian nurses, most of them had poor oral health practices [25, 35]. In terms of attitude, Indian and Australian nurses have been found to have a favorable attitude in this regard [35, 38]. It should be noted that differences in the demographic characteristics of the participants as well as the variety of data collection tools can make it difficult to compare the results of the studies. However, insufficient knowledge and practice of nurses about oral health especially toothbrush maintenance and use may be due to lack of continuous education. It seems that regular oral health training can improve nurses' awareness of their role as healthcare providers.

In the present study, no statistically significant relationship was found between age and nurses' knowledge, attitude, and practice. This finding is consistent with the findings of previous studies [10, 12, 25, 26, 29, 30, 34, 40]. However, Lin et al. reported a statistically significant relationship between nurses' age and their oral care practice [32]. The principles of oral hygiene, especially brushing and toothbrush maintenance, should be considered a health measure from childhood and be continued throughout life.

Consistent with previous studies [9–12, 25], in the present study, no statistically significant relationship was found between gender and nurses' knowledge, practice, and attitude. However, Baseer et al. reported a significant relationship between gender and nurses' practice. Having sufficient knowledge and practice about oral health including toothbrush maintenance and use should be considered by both sexes.

In keeping with previous studies [9, 10, 12, 23–25, 29], no statistically significant relationship was observed between the level of education and nurses' knowledge, practice, and attitude. However, in some studies, a statistically significant relationship has been reported [2, 11]. Nurses in every field of education are considered role models in society and should have a good attitude and sufficient knowledge and practice about oral health including toothbrush maintenance and use.

In the present study, there was no statistically significant relationship between work experience and the variables of knowledge, attitude, and practice of nurses. This finding is consistent with those of some previous studies [2, 23, 29, 33, 34, 40]. Due to the nature of the nursing profession, nurses with any work experience are expected to have sufficient knowledge and practice and a favorable attitude towards the principles of maintenance and use of toothbrushes.

### 4.1. Study Limitations

This study faced three limitations. First, data were collected through a self-report method, which could have affected the accuracy of the results. However, the researcher tried to increase the validity of the results by reassuring the participants of anonymity of questionnaires and visiting them at an appropriate time to complete the questionnaires. Second, due to the cross-sectional nature of the study, it was not possible to determine the causal relationships between demographic and knowledge, attitude, and practice variables. Third, due to the fact that different tools with different cutting points have been used in different studies, it can limit the accurate comparison of study results.

## 5. Conclusion

The results of this study indicated that nurses had moderate knowledge, attitude, and practice toward toothbrush maintenance and use, which does not seem acceptable. Since nurses are considered role models in society, they are required to have good knowledge, attitude, and practice regarding oral hygiene. Therefore, holding training courses on the principles of oral hygiene is recommended. It is also recommended to pay more attention to the issue of oral health, with emphasis on the maintenance and use of toothbrushes, in the nursing curriculum. Future studies are suggested to evaluate the factors related to nurses' knowledge, attitude, and practice regarding oral health and the effect of intervention measures on these variables.

## Figures and Tables

**Table 1:** Demographic characteristics of nurses.

	Variables	*n* (%)
Gender	Male	128 (39.4)
	Female	197 (60.6)
Education	BSc.^‡^	299 (92.0)
	MSc.^*∗*^	26 (8.0)
Age (years)	22–32	193 (59.4)
	33–42	124 (38.2)
	43–52	8 (2.5)
Work experience (years)	1–9	215 (66.2)
	10–18	102 (31.4)
	19–28	8 (2.5)

^‡^Bachelor of Science; ^*∗*^Master of Science.

**Table 2:** Nurses' knowledge, attitude, and practice scores in toothbrush maintenance and use.

Variables	Median (IQR^‡^)	Mean ± SD^†^
Knowledge	60.0 (20.0)	59.3 ± 16.4
Attitude	66.7 (33.3)	64.2 ± 20.7
Practice	50.0 (30.0)	51.5 ± 17.1

^‡^Interquartile range; ^†^standard deviation.

**Table 3:** Relationship between nurses' knowledge of toothbrush maintenance and use and demographic variables.

	Variables	Median (IQR^‡^)	Mean ± SD^†^	Test result
Gender	Male	60 (20.0)	58.1 (17.1)	*Z* = −1.20
	Female	60 (20.0)	60.0 (16.0)	*P*=0.228
Education	BSc.^‡^	60 (20.0)	58.9 (16.3)	*Z* = −1.24
	MSc.^*∗*^	60 (30.0)	63.8 (17.9)	*P*=0.213
Age (years)	22–32	60 (20.0)	58.9 (16.3)	*X* ^2^ = 1.47
	33–42	60 (20.0)	60.1 (17.0)	*P*=0.477
	43–52	50 (10.0)	55.0 (11.9)	
Work experience (years)	1–9	60 (20.0)	59.2 (15.9)	*X* ^2^ = 0.12
	10–18	60 (20.0)	59.4 (17.9)	*P*=0.942
	19–28	55 (17.5)	58.7 (11.2)	

^**‡**^Interquartile range; ^†^standard deviation; ^**‡**^Bachelor of Science; ^*∗*^Master of Science.

**Table 4:** Relationship between nurses' attitude of toothbrush maintenance and use with demographic variables.

	Variables	Median (IQR^‡^)	Mean ± SD^†^	Test result
Gender	Male	66.7 (33.3)	62.9 (21.7)	*Z* = −1.10
	Female	66.7 (33.3)	65.0 (19.9)	*P*=0.270
Education	BSc.^‡^	66.7 (33.3)	64.4 (20.3)	*Z* = −0.26
	MSc.^*∗*^	66.7 (33.3)	61.5 (24.8)	*P*=0.791
Age (years)	22–32	66.7 (25.0)	63.5 (20.5)	*X* ^2^ = 0.90
	33–42	66.7 (33.3)	65.3 (20.8)	*P*=0.635
	43–52	58.4 (41.7)	64.5 (24.3)	
Work experience age (years)	1–9	66.7 (33.3)	63.8 (20.7)	*X* ^2^ = 0.61
	10–18	66.7 (33.3)	65.2 (20.8)	*P*=0.737
	19–28	66.7 (16.7)	62.5 (19.4)	

^‡^Interquartile range; ^†^standard deviation; ^‡^Bachelor of Science; ^*∗*^Master of Science.

**Table 5:** Relationship between nurses' practice of toothbrush maintenance and use with demographic variables.

	Variables	Median (IQR^‡^)	Mean ± SD^†^	Test result
Gender	Male	50 (20.0)	49.4 (17.0)	*Z* = −1.94
	Female	50 (30.0)	52.8 (17.0)	*P*=0.052
Education	BSc.^‡^	50 (30.0)	51.9 (17.2)	*Z* = −1.47
	MSc.^*∗*^	40 (32.5)	46.9 (15.9)	*P*=0.140
Age (years)	22–23	50 (15.0)	51.9 (16.0)	*X* ^2^ = 0.93
	33–42	50 (30.0)	50.9 (18.7)	*P*=0.626
	43–52	40 (20.0)	47.5 (18.3)	
Work experience (years)	1–9	50 (20.0)	51.5 (15.9)	*X* ^2^ = 0.34
	10–18	50 (32.5)	51.7 (19.6)	*P*=0.842
	19–28	40 (20.0)	48.7 (12.5)	

^**‡**^Interquartile range; ^†^standard deviation; ^‡^Bachelor of Science; ^*∗*^Master of Science.

## Data Availability

The identified datasets analyzed during the current study are available from the corresponding author upon request.
